# Unfortunate Outcomes in Patients With SARS-CoV-2 Superimposed on Pneumococcal Pneumonia

**DOI:** 10.7759/cureus.10939

**Published:** 2020-10-14

**Authors:** Sherif Elkattawy, Ramez Alyacoub, Ahmed Mowafy, Islam Younes, Carlos Remolina

**Affiliations:** 1 Internal Medicine, Rutgers-New Jersey Medical School/Trinitas Regional Medical Center, Elizabeth, USA; 2 Internal Medicine, Trinitas Regional Medical Center, Elizabeth, USA; 3 Pulmonary and Critical Care Medicine, Trinitas Regional Medical Center, Elizabeth, USA

**Keywords:** covid-19 outbreak, pneumococcal antigen

## Abstract

The severe acute respiratory syndrome coronavirus 2 (SARS-CoV-2) and the disease it causes, coronavirus disease 2019 (COVID-19), continue to have socioeconomic as well as health implications worldwide. The virus has already led to over 200,000 deaths in the United States alone. This is most likely secondary to quick respiratory deterioration seen in patients inflicted with the virus. In other words, the heightened inflammatory response leads to major organ system damage, which leads to rapid decompensation of the patient's clinical condition. Interestingly enough, some patients present with both the novel virus as well as a superimposed bacterial infection that further complicates the management of the disease. We present a case of a patient with a positive polymerase chain reaction (PCR) test for SARS-CoV-2 as well as a pneumococcal urine antigen; he was treated with both appropriate antibiotics as well as dexamethasone and remdesivir for pneumonia and novel virus, respectively. The patient's hypoxemia continued to worsen with appropriate means of oxygenation and eventually led to cardiac arrest.

## Introduction

Coronavirus disease 2019 (COVID-19), caused by the severe acute respiratory syndrome coronavirus 2 (SARS-CoV-2), has resulted in a worldwide pandemic that continues to overwhelm healthcare systems and economies. The virus can manifest in a wide range of presentations, from asymptomatic infections to severe acute respiratory syndrome requiring mechanical ventilation. Like in other respiratory virus infections, bacterial superinfections and coinfections have been reported in COVID-19 as well. Streptococcus pneumonia is the most common cause of community-acquired pneumonia (CAP). Identifying bacterial superinfections and coinfection in patients with COVID-19 infection can be difficult due to overlapping presentations, which in turn affect the management of these patients. We present a case of a patient who presented with fever and respiratory symptoms and was found to have pneumococcal pneumonia as well as COVID-19 infection.

## Case presentation

**Figure 1:**
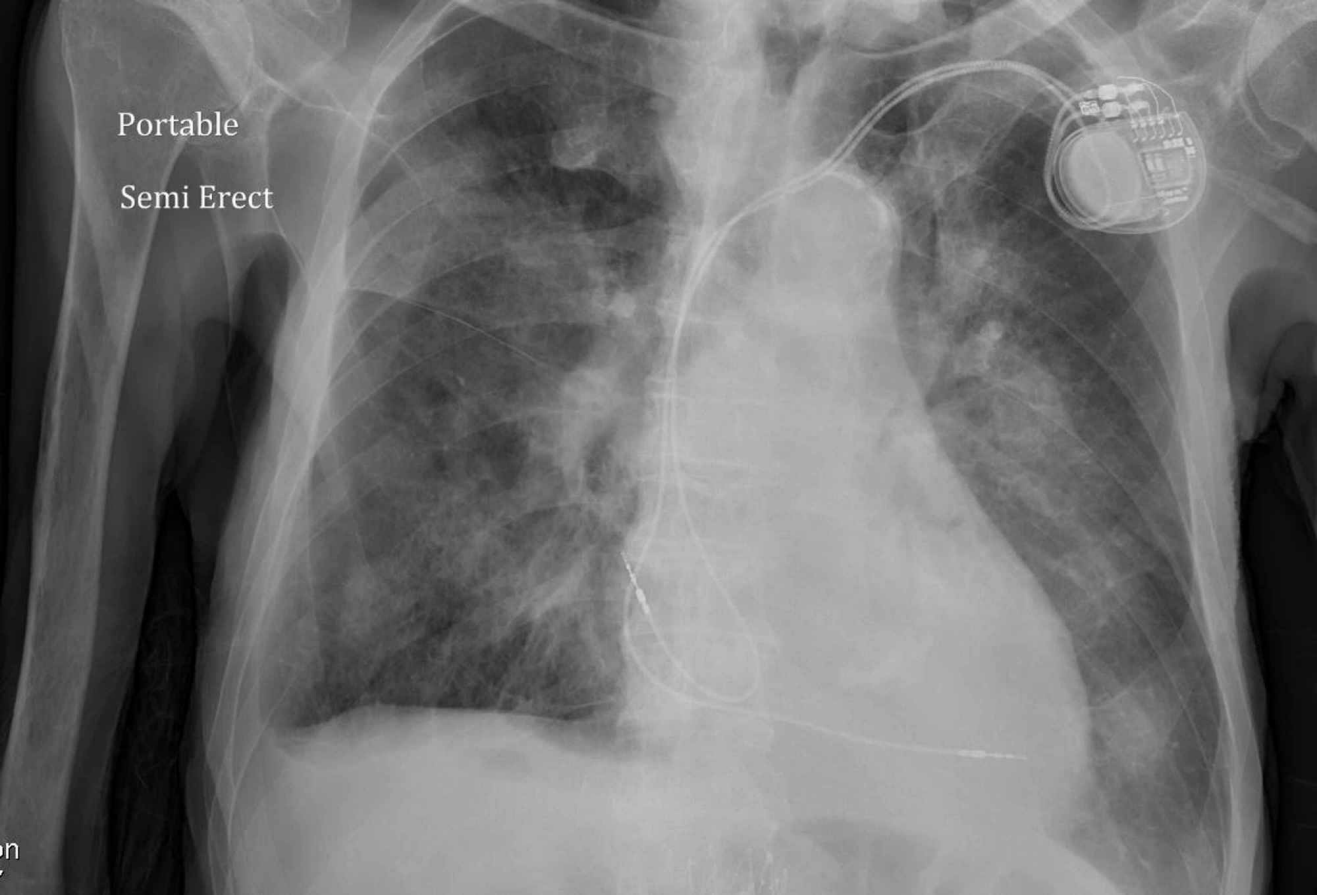
Chest X-ray showing bilateral lower lobe infiltrates

**Figure 2:**
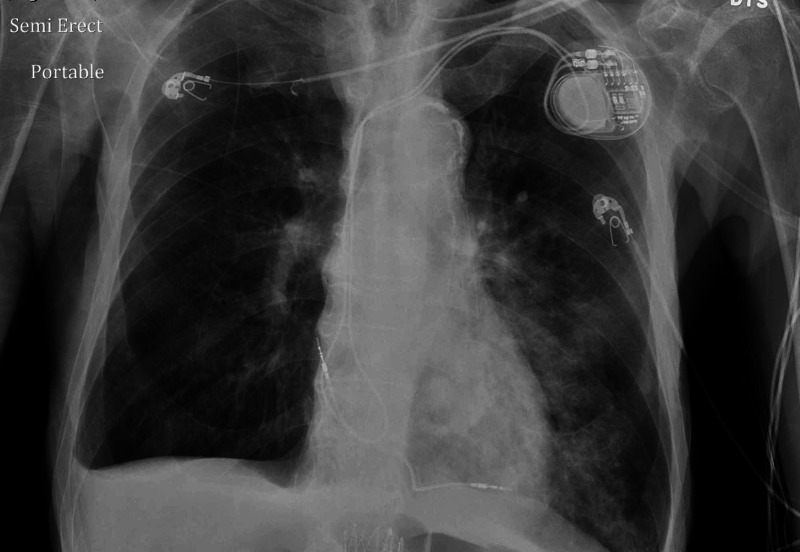
Chest X-ray showing left lower lobe infiltrates

**Figure 3:**
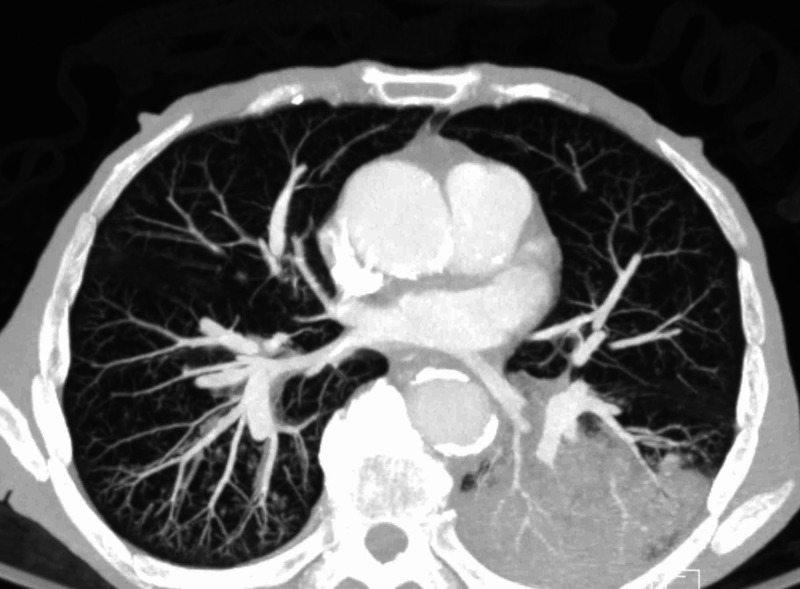
CT angiogram of the chest shows significant left lower lobe infiltrate CT: computed tomography

An 88-year-old male patient with a past medical history of dementia, coronary artery disease, repaired abdominal aortic aneurysm, dyslipidemia, spinal stenosis, and osteoarthritis presented with cough and shortness of breath of unclear duration. He was found on admission to be febrile, hypoxic, with oxygen saturation in the low 80s on room air, hypotensive, with sinus tachycardia. Initial chest X-ray showed bilateral lower lobe infiltrates as seen in Figure 1. A repeat chest X-ray two days later showed more infiltrates on the left lower lobe as seen in Figure 2. A CT chest angiogram showed significant left lower lobe infiltrate as seen in Figure 3.

Labs were remarkable for marked leukocytosis (50 K/UL; normal range: 4.8-10.8 K/UL). The patient was then admitted to the ICU. He tested positive for pneumococcal antigen and COVID-19 polymerase chain reaction (PCR) test. The patient was found to be clinically improving on broad-spectrum antibiotics and supportive therapy. He was downgraded to the medical floors on oral antibiotics and nasal cannula 2 LPM saturating 95%. Two days later, the patient started desaturating again and spiking fevers of 100.5 °F. He was then started on remdesivir and dexamethasone for COVID-19 pneumonia based on the data available at that time. The patient drastically deteriorated and was placed on bilevel positive airway pressure (BiPAP). Unfortunately, he went into cardiac arrest secondary to hypoxia, and a code blue was called. Advanced cardiovascular life support (ACLS) was performed; however, we were unable to achieve the return of spontaneous circulation (ROSC).

## Discussion

The SARS-CoV-2 virus, which causes COVID-19, started as a cluster in China and has turned into a worldwide pandemic. COVID-19 patients usually present with fever, cough, sore throat, and shortness of breath. However, asymptomatic infections have been described as well, though their frequency is still unknown. On the other hand, symptomatic infections can range from mild with minimal or no pneumonia to severe, characterized by 50% lung involvement on imaging, to even critical disease with multiorgan involvement [1].

Bacterial infections have been well described in the context of viral respiratory infections, including influenza, SARS, and Middle East respiratory syndrome (MERS). The bacterial infection is divided into coinfections that occurs in the initial phase of a viral infection or secondary bacterial infection during the recovery phase [2]. Secondary bacterial infections have been reported as a common complication with COVID-19 infection. In a meta-analysis by Langford et al., bacterial coinfection was reported in 3.5% of patients and secondary infection in 14.3% of patients with COVID-19 [3]. Secondary bacterial infections are also common in mechanically ventilated patients and usually present as similar to hospital-acquired pneumonia.

Diagnosing secondary bacterial infections and coinfections can be challenging. In patients with concomitant viral infections, specifically in COVID-19 patients, deterioration of the respiratory status of the patient can be a sign of the progression of the COVID-19 or secondary bacterial infection. Additional findings that increase the likelihood of bacterial infection and indicate empiric antibiotic therapy prior to getting culture results include new leukocytosis, particularly neutrophilic leukocytosis, new fever, and worsening respiratory failure [4].

Streptococcus pneumonia has traditionally been the most common cause of CAP. However, the frequency of pneumococcal pneumonia has been decreasing as a result of the use of pneumococcal vaccines and herd immunity [5]. Risk factors for pneumococcal pneumonia include cigarette smoking, chronic obstructive pulmonary disease, and viral infections. CAP is usually diagnosed based on patient history and X-ray findings. It is usually treated empirically. However, isolation of the causative agent can help direct antibiotic therapy. Streptococcus pneumonia can be identified in sputum or blood culture; detection of pneumococcal antigen in the urine is more sensitive. Procalcitonin has not been found to be helpful in pneumococcal pneumonia [6]. Chest X-ray typically shows lobar infiltrate, which is different from the typical COVID-19 findings of bilateral, peripheral, and lower-zone-predominant air-space disease.

Treatment of pneumococcal pneumonia is by antibiotics. An early diagnosis of coinfection is required. Future studies on concomitant bacterial infection in COVID-19 are needed, especially focusing on the temporal relationship of bacterial infection relative to the patient presented as well as accurate diagnosis. Our patient likely had coinfection of pneumococcal pneumonia along with early COVID-19 infection. Although he was treated for pneumococcal pneumonia, his COVID-19 progressed, leading to respiratory failure and death.

## Conclusions

SARS-CoV-2 has resulted in over 200,000 deaths in the United States. Understanding whether a patient is infected solely by the virus or if there is a superimposed bacterial infection as well is crucial to the management of these patients. This study discussed the case of an unfortunate patient who was treated appropriately for pneumococcal pneumonia and COVID-19; however, his respiratory status deteriorated during the hospital course. We urge our fellow researchers to conduct prospective cohort studies with regard to the optimal treatment of concomitant novel coronavirus and superimposed bacterial infection.
